# Bioimpedance as a measure of fluid overload in patients recently admitted to intensive care

**DOI:** 10.1186/cc14268

**Published:** 2015-03-16

**Authors:** M O'Connor, E Galtrey, CJ Kirwan, JR Prowle

**Affiliations:** 1 Barts Health NHS Trust, London, UK

## Introduction

Fluid overload is associated with adverse outcomes in critical illness; however, better methodology is required for its quantification. Bioelectrical impedance analysis (BIA) represents a noninvasive method for quantification of fluid overload [1], but has not been widely taken up in the ICU.

## Methods

We assessed changes in fluid balance and performed daily BIA (using a Maltron BioScan 920-II; Maltron International Ltd, UK) over 3 days in consecutive ICU admissions with LOS >72 hours.

## Results

**Figure 1:**
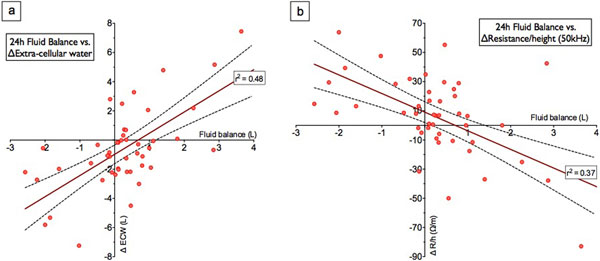
**Change in BIA-measured ECW (a) or R/h at 50 kHz (b) versus daily fluid balance**.

Of 24 patients 71% were male, median age was 65 years and APACHE II score was 15. Eleven patients had a medical diagnosis and 13 a surgical or trauma reason for admission. Seventy-one percent were mechanically ventilated and 67% were on vasopressors or inotropes. Median BIA-estimated extracellular water was 25.2 l (IQR 22 to 28) on day 1, equating to excess fluid of 7.2 l (IQR 5 to 13.9). Median right body resistance normalized to height at 50 kHz (R50/h) on day 1 was 214 Ω/m (IQR 187 to 256). Daily change in ECW and R50/h correlated with daily fluid balance between BIA measurements (*R*^2 ^= 0.48 and 0.37 respectively) (Figure 1).

## Conclusion

BIA suggests many patients already have significant fluid overload on the first day of ICU admission. Overall, changes in device-specific algorithms for ECW estimation and measured resistances correlated with recorded fluid balance; however, there were inconsistencies in the number of individual patients. Prospective assessment is required to establish whether BIA measurements can be used to assist fluid management in the ICU.
